# Animal model of acute gout reproduces the inflammatory and ultrasonographic joint changes of human gout

**DOI:** 10.1186/s13075-015-0550-4

**Published:** 2015-02-26

**Authors:** Carlos Pineda, Arturo J Fuentes-Gómez, Cristina Hernández-Díaz, Yessica Zamudio-Cuevas, Javier Fernández-Torres, Ambar López-Macay, Ismael Alba-Sánchez, Javier Camacho-Galindo, Lucio Ventura, Luis E Gómez-Quiróz, María Concepción Gutiérrez-Ruíz, Francisco García-Vázquez, Anthony M Reginato, Marwin Gutiérrez, Alberto López-Reyes

**Affiliations:** Molecular Synovioanalysis Laboratory, Musculoskeletal Ultrasound Laboratory, Hip-Knee Joint Reconstruction Department, Instituto Nacional de Rehabilitación, Calzada Mexico-Xochimilco 289, Tlalpan, 14389 Mexico City, Mexico; Departamento de Ciencias de la Salud, Universidad Autónoma Metropolitana, Avenida San Rafael Atlixco 186, Iztapalapa, 09340 Mexico City, Mexico; Pathology Department, National Institute of Pediatrics, Av. Insurgentes Sur 3700-C, Coyoacán, 04530 Mexico City Mexico; Rheumatology Division, Rhode Island Hospital, The Warren Alpert Medical School of Brown University, 593 Eddy Street, Providence, 02903 RI USA; Clinica Reumatologica, Università Politecnica delle Marche, Via dei Colli 52-60035, Jesi, Ancona, Italy; Biological and health sciences PhD program, Universidad Autónoma Metropolitana, Avenida San Rafael Atlixco 186, Iztapalapa, 09340 Mexico City Mexico; Molecular Microbiology Laboratory, Department of Microbiology, Escuela Nacional de Ciencias Biológicas (ENCB), Instituto Politécnico Nacional (IPN), Prolongación de Carpio y Plan de Ayala S/N, Col. Casco de Santo Tomás, Miguel Hidalgo, 11340 Mexico City, Mexico

## Abstract

**Introduction:**

Gout is an inflammatory condition induced by the deposition of monosodium urate (MSU) crystals in the joints and soft tissues that can produce acute or chronic arthritis. Several animal models of crystal-induced inflammation have been proposed that involve direct injection of MSU-crystals into different anatomical structures; however, only a few of these models reflect a true diarthrodial joint microenvironment in which an acute gouty attack takes place. The aim of this study was to assess the inflammatory and structural joint changes in a rabbit model of acute gout attack by ultrasound (US), synovial fluid (SF) and histopathological analyses.

**Methods:**

Under US guidance, 42 rabbit knees were randomly injected with a suspension of 50 mg/ml of either MSU or allopurinol synthetic crystals. The control group received intra-articular vehicle of phosphate-buffered saline (PBS). US evaluation, SF and histopathological analyses were performed at days 1, 3, and 7.

**Results:**

A total of 21 rabbit knees were assigned to the control group, 12 to the MSU-crystals group, and 9 to the allopurinol crystals group. By US, the MSU crystals group displayed the double contour sign and bright stippled aggregates in 67% and 75% of joints, respectively. Neither control knees nor allopurinol crystals group displayed these US signs. Power Doppler (PD) signal was moderate to intense in the MSU-crystals group and greater than both the allopurinol crystal and control groups at day 1 (*P* <0.001) and 3 (*P* <0.05), with its practical disappearance by day 7. SF leukocyte count was 40,312 ± 6,369 cells/mm^3^ in the MSU-crystals group, higher than in controls (*P* = 0.004) and allopurinol crystal group (*P* = 0.006). At day 7, SF leukocyte count decreased in both MSU and allopurinol crystal groups reaching the non-inflammatory range. Histologically, at day 3 intense synovial polymorphonuclear cells infiltration and MSU aggregates were identified.

**Conclusion:**

The rabbit model of MSU crystal-induced acute arthritis efficiently reproduces the inflammatory, US, SF and histopathological changes of the human acute gouty attack.

**Electronic supplementary material:**

The online version of this article (doi:10.1186/s13075-015-0550-4) contains supplementary material, which is available to authorized users.

## Introduction

Gout is an inflammatory condition induced by the deposition of monosodium urate (MSU) crystals in joints and soft tissues that can produce an acute or chronic arthritis [1]. The early phase of MSU-induced inflammation is associated with activation of the NACHT, LRR and PYD domain-containing protein 3 (NALP3) inflammasome or cryopyrin, triggering the release of IL-1β and IL-18 [2], thus promoting cellular activation, cytokine and chemokine production, and infiltration of neutrophils, leading to an acute gouty attack [3,4]. Despite their similar morphology, allopurinol crystals have not been found to activate the NALP3 inflammasome [5].

Several animal models of crystal-induced inflammation have been studied that involve direct injection of MSU crystals into both intra-articular and extra-articular locations. Extra-articular locations include: intradermal, subcutaneous, and intraperitoneal areas; however, only the intra-articular model reflects the true diarthrodial joint microenvironment, in which an acute gouty attack takes place [6-16].

Imaging techniques such as ultrasound (US) may reinforce the diagnosis, monitoring, and management of gout [17-19]. Several studies have shown its capability to detect both intra- and extra-articular abnormalities in patients with gout [20-28]. The high reflectivity of MSU-crystal aggregates, and the ability of US to detect even minimal MSU-crystal deposits explain the high sensitivity of US for revealing MSU deposits [26]. To our knowledge no animal model has been used to sequentially evaluate the changes of MSU crystal-induced acute arthritis, using a multimodality approach including US as an imaging tool. Thus, the aim of this study was to assess the spatial and temporal joint changes in a rabbit model of acute MSU crystal-induced arthritis by US, synovial fluid (SF) cell counts, and histopathological analyses.

## Methods

### Study subjects

A total of 21 male adult white New Zealand rabbits weighing 2 kg, were acclimatized to the animal facility environment during 1 week prior to the study. The rabbits were housed with a standard diet, and circadian rhythm synchronized with a 12-h:12-h light-dark cycle. This protocol was approved by the Ethics and Research Committee of the *Instituto Nacional de Rehabilitación* (reference EP/01-12), and experiments were performed in accordance with current ethics guidelines and the Institutional Animal Care and Use Committee (reference BIO/01/12). All procedures were in accordance with the guidelines of the Official Mexican Standard NOM-062-ZOO-1999 [29].

### Synthetic MSU and allopurinol crystal preparation

MSU and allopurinol crystals were prepared using the Denko and Whitehouse method [30] modified by Scanu *et al*. [3]. Crystals were sterilized at 180°C and suspended in sterile PBS at a concentration of 50 mg/ml. The morphological and birefringence properties of MSU and allopurinol crystals were assessed by standard light, polarized light and scanning electron microscopy (Additional file 1). The MSU crystals were uniform in size (12 ± 2 μm in length), needle-shaped, and exhibited negative birefringence. The absence of microbial contaminants was confirmed by negative cultures for microorganisms and were bacterial endotoxin-free (<0.01 EU) by the *Limulus* amebocyte cell-lysate assay (Sigma®, St Louis, MO, USA).

### Animal model and study design

**Figure 1:**
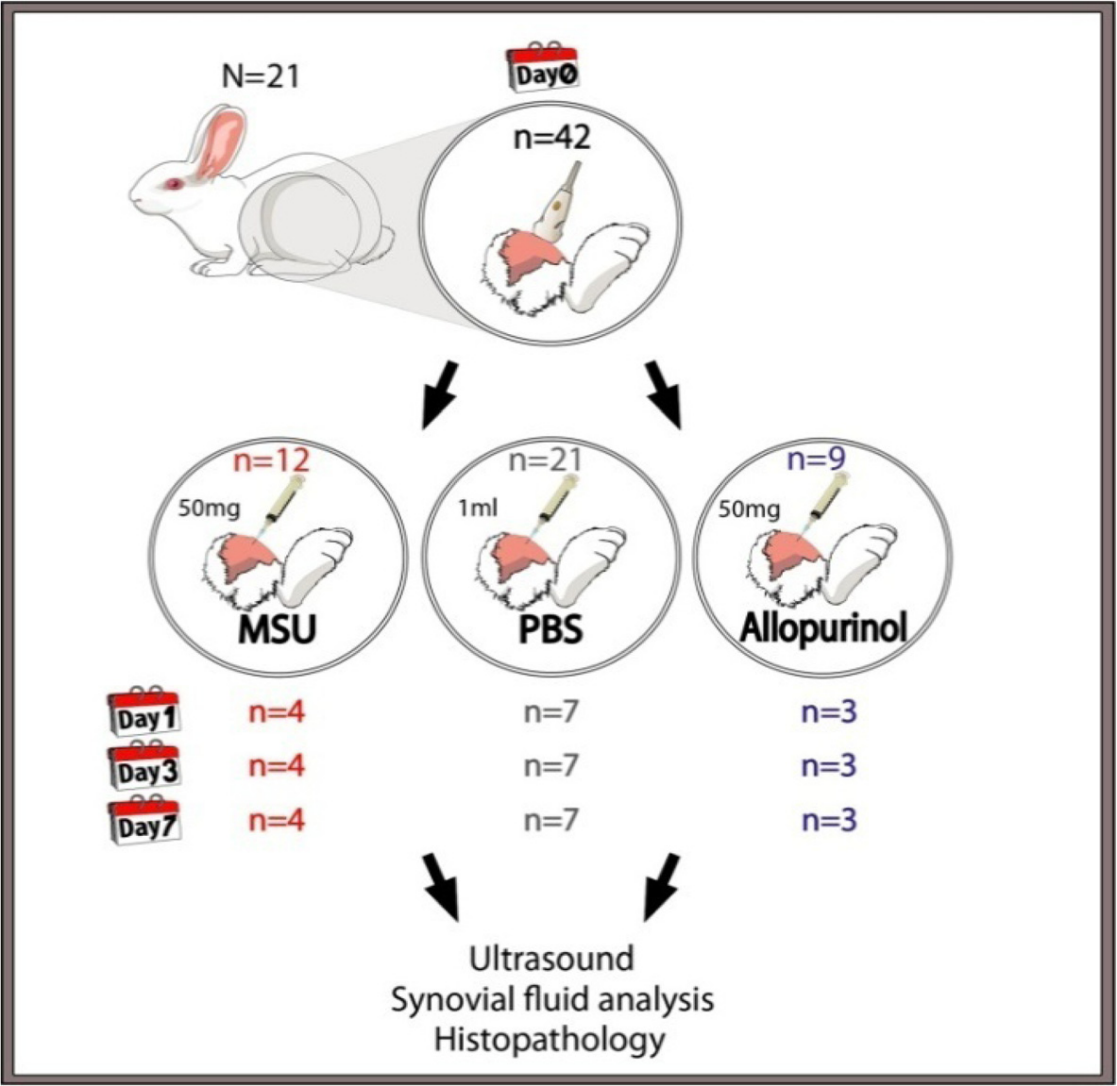
**Schematic diagram of the study protocol. MSU, monosodium urate.**

At baseline (day 0), US examinations of 42 rabbit knee joints were performed. Each animal was then sedated with 6.5% pentobarbital sodium solution intravenously. Under US guidance, one knee was intra-articularly injected with 1 ml of PBS (control group), while the contralateral joint was randomly injected with 1 ml of a suspension containing 50 mg/ml of either MSU or allopurinol crystals (Figure 1). At days 1, 3, and 7, US scans, US-guided arthrocentesis, and SF analysis were obtained from all injected joints. At the end of each time point, animals were euthanized and tissue samples were obtained for histological analysis.

### Ultrasound assessment and interpretation

US scans were performed using a MyLab25® device (Esaote Biomedica, Genoa, Italy) equipped with a high-frequency (10 to 18 MHz) linear array transducer. The power-Doppler (PD) technique was used to detect blood flow. PD settings included: pulse repetition frequency of 700 Hz, Doppler frequency of 7.1 MHz, low wall filter, and Doppler gain adjusted to avoid random noise visualization. All US examinations were performed by two experienced sonographers (CHD and LV), who were blinded to the injection type at all time points. Before the study, the sonographers reached consensus on the scanning technique to adopt and the gout-related US findings to evaluate. Rabbit knees were scanned using a multiplanar technique. The insonation angle was adjusted in order for it to be perpendicular to the cartilage surface. B-mode gain was initially set in order to obtain maximal contrast among tissues and was successively reduced to its lowest level, allowing only visualization of hyperechoic structures, using the bony cortex as reference.

The presence of US-defined synovial effusion and synovial hypertrophy was based on Outcome Measures in Rheumatology (OMERACT) definitions [31]. The presence of intra-articular PD signal was graded on a semiquantitative scale (0 to 3) as previously described [32]. The following US features of MSU-crystal deposition were assessed: the double contour sign (DCS) and bright stippled aggregates (BSA), as they were previously described as among the most frequently identified elementary lesions of gout [27]. The DCS is defined as an abnormal hyperechoic band over the superficial margin of the articular cartilage. To further distinguish DCS from the cartilage interface sign, dynamic testing was performed. BSA is defined as intra-articular heterogeneous hyperechoic foci with or without posterior shadowing over a hypo- or anechogenic background. Finally, all US images were interpreted in conjunction with a third, blinded, experienced sonographer (CP). Discrepancies were resolved by consensus among the three sonographers.

### Synovial fluid analysis

At days 1, 3, and 7, US-guided arthrocentesis of both control and crystal-injected knees were performed [33]. SF aspirates were evaluated under standard light and polarized light microscopy to assess the presence, morphology, and birefringence of crystals. SF leukocyte count was performed using a Neubauer chamber.

### Histological assessment

Histological evaluation was performed at days 1, 3, and 7. All knee joints were dissected, a gross pathological examination was carried out and then fixed in 100% alcohol for 24 h; then decalcified in 5% nitric acid-ethanol for 48 h, and embedded in paraffin. Tissue sections (4 μm) were stained with H&E to assess inflammatory cell infiltrates; safranin-O dye was used to assess cartilage proteoglycan content, and eosin Y solution 0.5% alcohol, to prevent the dissolving of MSU crystals and preserve their birefringence properties [34,35].

### Statistical analysis

Data were expressed as mean ± standard error of the mean (SEM). Statistical significance between the groups was assessed by two-tailed analyses using GraphPad Prism 6.0 for Windows. Frequencies and proportions were expressed as percentages to describe categorical data, and differences were analyzed using the Fisher exact test. The Mann-Whitney *U*-test and two-way analysis of variance were computed for continuous variables. A *P*-value <0.05 was considered significant.

## Results

Twenty-one knee joints were assigned to the control group, and randomization resulted in a total of 12 knees in the MSU-group and nine in the allopurinol crystals group (Figure 1).

### Ultrasound findings

**Figure 2:**
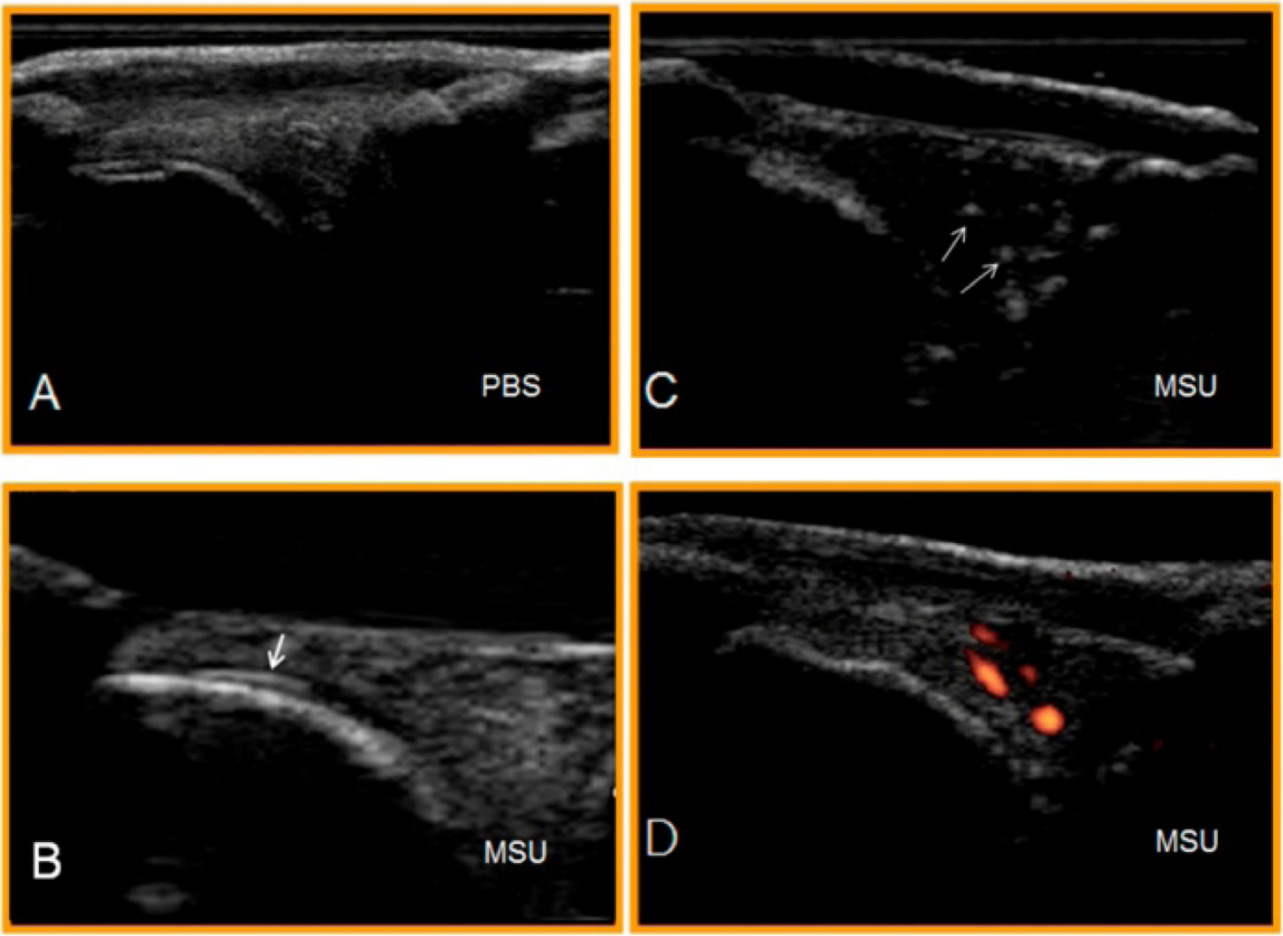
**Longitudinal ultrasound images of the rabbit knee joints at day 1. (A)** Control group showing no abnormalities. **(B,** **C, D)** Representative images of the monosodium urate (MSU)-crystal group: **(B)** double contour sign (DCS) (arrow); **(C)** bright stippled aggregates (BSA) (arrows); **(D)** intra-articular power-Doppler signal.

At baseline prior to joint injections neither US gray-scale abnormalities indicating MSU deposition nor intra-articular PD signals were detected. All intra-articular knee injections were successfully carried out under US guidance. In the MSU-crystals group DCS was present in 8 of 12 (67%) knee joints. Chronologically, the DCS sign appeared as early as day 1 in two of the four evaluated knees; at day 3 it became apparent in three more knees, and finally at day 7 in three additional knees. The BSA was found in 9 of the same 12 (75%) MSU-injected knee joints which contemporaneously exhibited this US sign (Figure 2). Neither control knees nor allopurinol crystal group showed either DCS or BSA sonographic signs.

**Figure 3:**
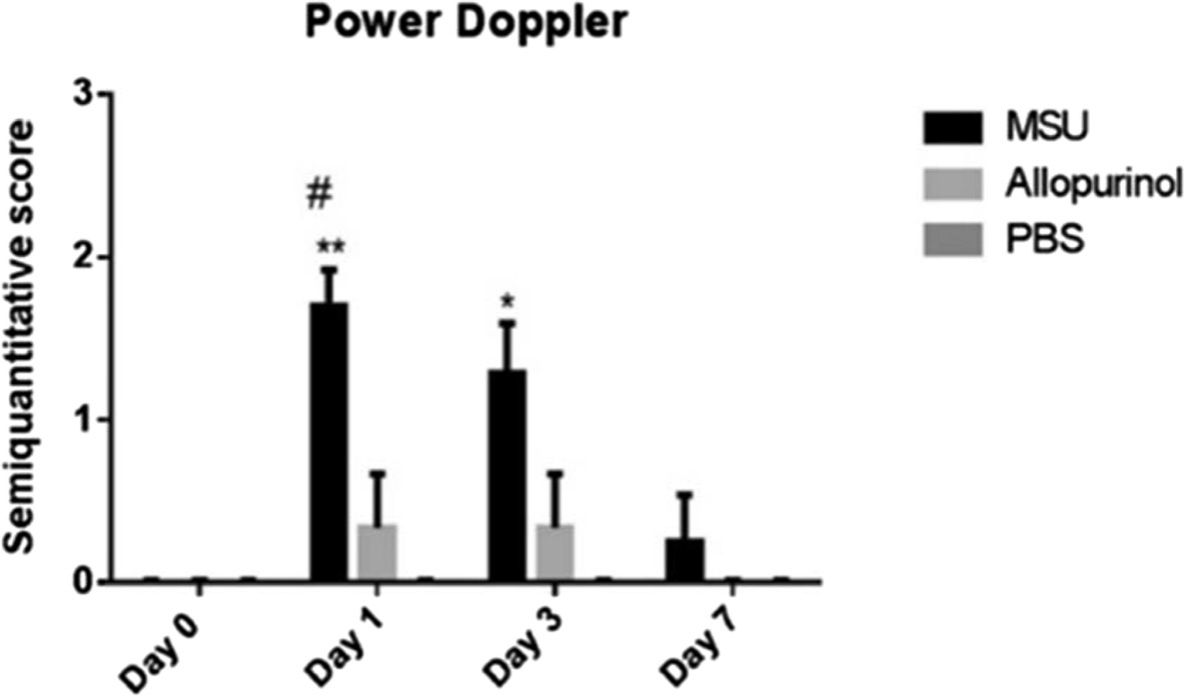
**Semiquantitative power-Doppler (PD) score for rabbit knees injected with monosodium urate (MSU), allopurinol and control groups.** At days 1 and 3 an increase in PD signal intensity in the MSU crystal group was found when compared to the control group (**P <0.01 and *P <0.05, respectively). Additionally, a significant increase of PD signal was present when comparing the MSU to the allopurinol crystal groups (^#^ *P* <0.05).

Regarding intra-articular blood flow, a moderate to intense increase in synovial vascularity was observed in the MSU-crystal group; PD signal intensity was greater compared to the control group at days 1 (*P* <0.01) and 3 (*P* <0.05). At day 7 a decrease in synovial perfusion (PD signal intensity) was observed in the MSU-crystal group (Figure 3). When comparing MSU-injected joints to those of the allopurinol crystal group, a significant increase in PD signal intensity in the MSU group was also found at day 1 (*P* = 0.026).

### Synovial fluid analysis

**Figure 4:**
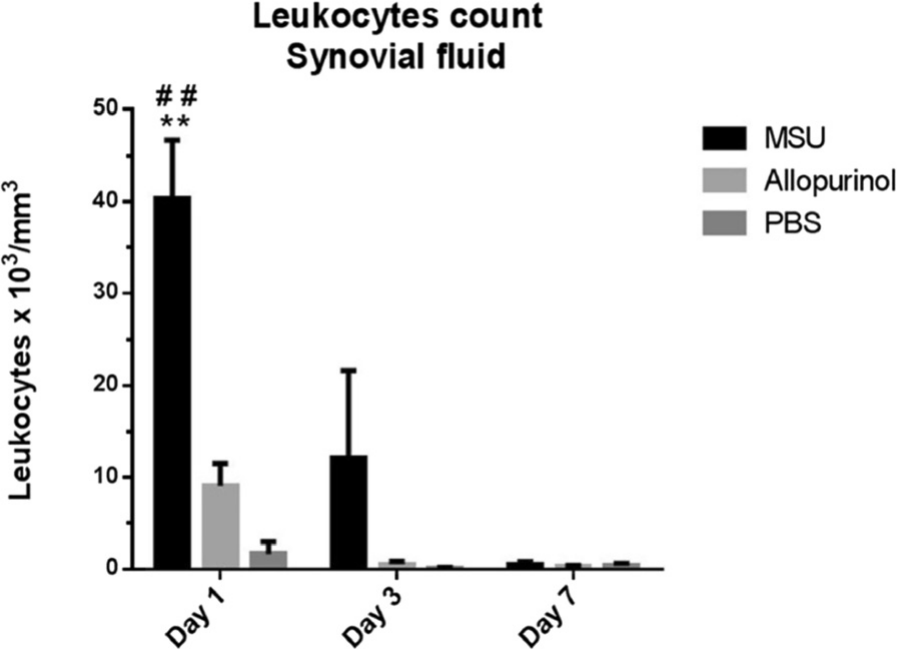
**Synovial fluid leukocyte cell count from rabbit knees in the monosodium urate (MSU), allopurinol and control groups.** A significant increased cell count was present at days 1 and 3 in the MSU-crystal group when compared to PBS (**P <0.01) and allopurinol crystal groups (^##^ *P* <0.01).

**Figure 5:**
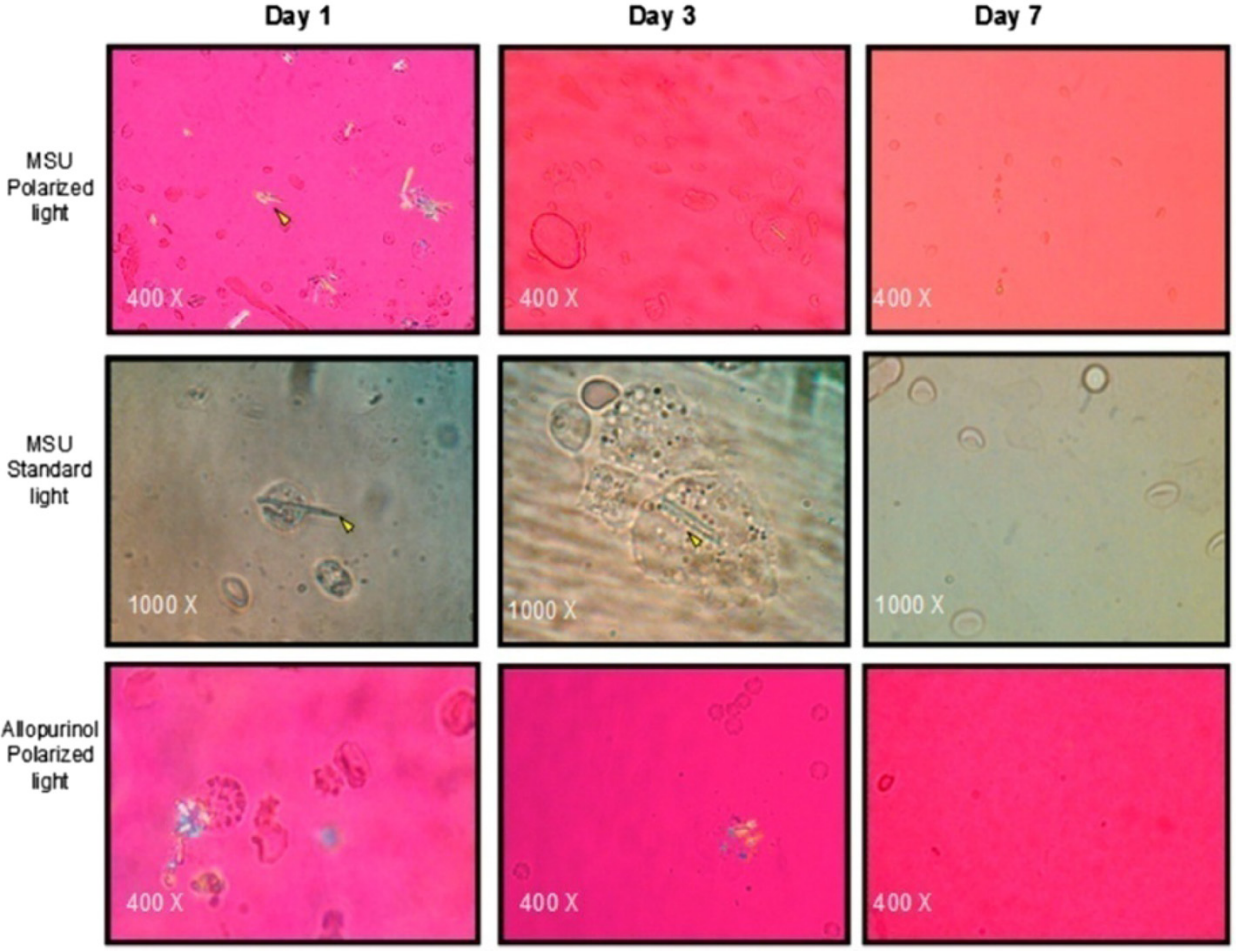
**Standard light and compensated polarized light microscopic analysis of synovial fluid (SF) from the monosodium urate (MSU) and allopurinol groups.** Observe the presence of intra- (arrows) and extra-cellular negatively birefringent needle-shaped crystals in the MSU-crystal group at day 1. MSU crystals phagocytized by polymorphonuclear cells were seen at days 1 and 3. Absence of MSU and allopurinol crystals was observed at day 7.

SF aspirates at day 1 revealed the presence of numerous negatively birefringent needle-shaped crystals in the MSU-crystal group; the majority of crystals were extracellularly located while a minority were phagocytized by polymorphonuclear cells. Mean white-cell count in the MSU-crystal group was 40,312 ± 6,369 cells/mm^3^; this cell count was significantly higher than in the control group (1,687 ± 1,303 cells/mm^3^; *P* = 0.004) as well as in the allopurinol-crystal group (9,050 ± 2,477 cells/mm^3^*; P* = 0.006). However, when comparing SF cell counts from the allopurinol-crystal group with those of the control group, there was no significant difference. In comparison to day 1 the SF cellular content from both the MSU and allopurinol crystal groups decreased at day 3 to 12,075 ± 9,545 and 605 ± 75 cells/mm^3^, respectively. It was noteworthy that at day 3, SF from the MSU-crystal group displayed giant multivacuolated cells [36], cellular debris, crystal fragments phagocytized by polymorphonuclear cells, and scarce extracellular crystals. At day 7, SF leukocyte counts decreased in both the MSU and allopurinol crystal groups within the non-inflammatory range (to 566 ± 300 cells/mm^3^ and 316 ± 145 cells/mm^3^, respectively), and no crystals were observed by polarizing microscope examination (Figures 4 and 5).

### Histological analysis

**Figure 6:**
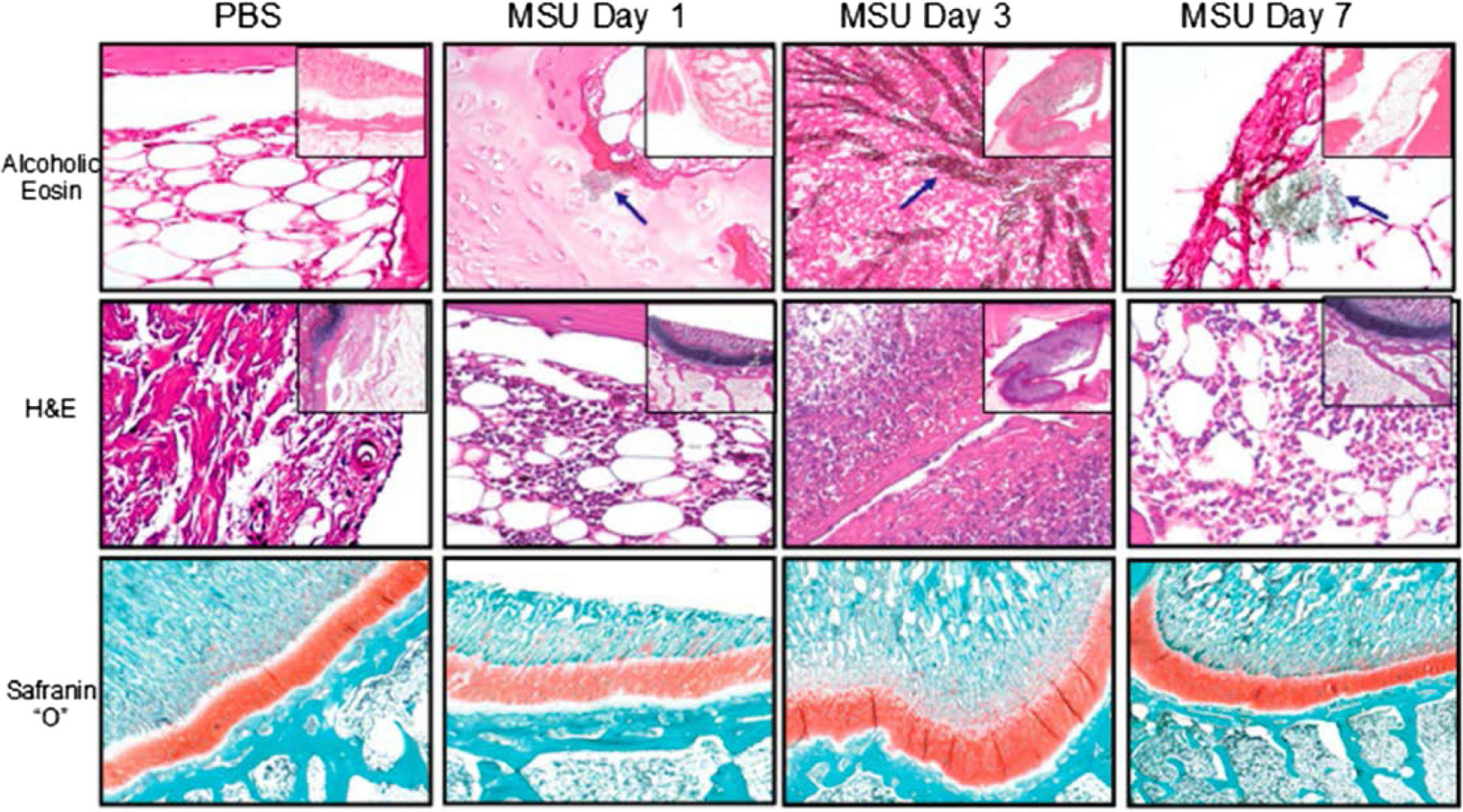
**Histopathological analysis of rabbit knee joint injected with monosodium urate (MSU), allopurinol and control groups.** Hypertrophy, inflammatory cell infiltration and MSU crystal aggregates (arrows) were present in the synovium starting from day 1. No cartilage abnormalities were observed at any time points.

Evaluation of the gross anatomic examination of the MSU-crystal injected knee joints showed MSU crystals forming white aggregates on the surface of the articular cartilage. Histological analysis of the MSU-crystal group showed focal synovial hyperplasia, mild reactive changes, vessel congestion, and patchy acute inflammatory cellular infiltrates at day 1. In addition scarce synovial membrane microcrystalline aggregates were observed (Figure 6). By day 3 a thickened and hyperplastic synovium, large synovial MSU-crystal clusters, and intense polymorphonuclear cell infiltration, in addition to some plasma cells and macrophages, were identified. At day 7 the MSU-crystals group showed scattered, small-sized microcrystalline aggregates and focal neutrophilic infiltrates consistent with mild and focal acute synovitis. Evaluation of cartilage proteoglycans showed no abnormalities in the content and distribution of sulfated proteoglycans in any of the study groups. No histological abnormalities were observed in the allopurinol crystal or control groups. However, histological sections of the articular cartilage failed to demonstrate deposition of MSU-crystals as seen in the gross anatomical specimens.

## Discussion

This study sequentially evaluated the temporal and spatial ultrasonographic, inflammatory, and structural joint changes observed in an animal model of acute gouty attack, by applying a multimodality approach.

In the past, a variety of animal models have been used to study the acute inflammatory response in crystal-induced arthritis [10-13]. Faires and McCarty reproduced an acute gouty episode by injecting 20 mg of MSU crystals into their own knees and into a canine animal model [14]. Subsequently, murine models of peritoneal [7,11] and subcutaneous air-pouch cavity of MSU-crystals have been utilized to study the inflammatory response [7,18]. Torres *et al*. [37] evaluated behavioral changes in mice after an intra-articular injection of MSU crystals and quantified systemic inflammatory biomarkers. An adult Japanese female rabbit model was used by Nishimura *et al*. [38] to evaluate the role of locally produced IL-8 in knee joints injected with MSU crystals.

On the other hand, contrast-enhanced US has been employed to assess synovial vascularity in a rabbit knee model of carrageenan-induced inflammatory arthritis [39]. To the best of our knowledge there are no animal models that reported the use of US guidance to ensure proper needle placement in their very small joint cavities, allowing successful joint aspirations and injections, thus avoiding damage to adjacent anatomic structures.

Using a multimodality approach to monitor the temporal and spatial sonographic, inflammatory, and morphostructural changes observed in a rabbit knee model of acute gouty arthritis, we were able to reproduce two highly specific US signs of human gout: DCS and BSA [17,23,27,40]. Our results provide gross pathological evidence that the development of the characteristic sonographic features of gout, DCS and BSA after synthetic MSU-crystal injection, resulted from the precipitation of MSU crystals on the superficial layer of the articular cartilage and intra-articular soft tissues, respectively.

On the other hand, allopurinol crystals were used as a negative, microcrystalline, control group, because previous reports have shown the failure of these crystals to activate the NALP3 inflammasome [5,41]. US failed to demonstrate the presence of DCS and BSA in the allopurinol crystal-injected animal. However, our results at the joint level, showed a low-grade, limited, and transient inflammatory response demonstrated by mild intra-articular PD signal plus an inflammatory range of SF leukocyte cell counts.

By means of a multimodal approach, several temporal and spatial, early, MSU-deposition, gouty changes were observed in the rabbit knee model. DCS appeared in one half of MSU-injected joints at day 1, and increased up to 75% at days 3 and 7, indicating that these crystals tend to deposit very early in the initial acute, inflammatory stages of disease, and not only in its late, chronic stages, as previously proposed [1]. As expected, from the synovial vascularization perspective, PD signals appeared as early as day 1, revealing hyperemia of synovial tissues in acutely inflamed rabbit joints, followed by a gradual reduction in signal intensity at days 3 and 7, respectively. These findings are consistent with the human counterpart; as atypical acute gouty attack is self-limiting, it peaks at 24 to 48 h after symptoms appear, and lasts 5 to 10 days [4]. US evaluations in the rabbit model failed to show additional, previously reported, sonographic findings in gouty patients, such as tophus, hyperechoic cloudy areas, and/or erosions [27]. The absence of these findings in our acute inflammatory animal model suggests that an extended time period with MSU crystals may be required to produce and display these elementary lesions as seen in gout on US.

A temporal correlation between SF cell counts and US findings was found at all experimental study time points. In contrast, histological findings were delayed in time relative to the SF inflammatory changes. Total SF cell counts from the MSU crystal group reached an inflammatory range that peaked at day 1. Despite the lack of standard normal values reported for rabbit SF, the control group displayed cell counts <2,000 cells/mm^3^ that in its human counterpart are considered non-inflammatory [42]. At day 1 pleomorphic cells, consistent with macrophages, were observed. These resident macrophages and infiltrating monocytes are responsible for initiating and driving the early inflammatory phase of the acute gout attack [7,43,44]. Histological assessment revealed marked synovial lining hyperplasia following injection of MSU crystals at days 3 and 7. Remarkably, only scattered inflammatory cells were observed infiltrating the synovium of the MSU crystal group at day 1; notwithstanding this, the number of inflammatory cells in the SF reached its peak. However, intense infiltration of the synovial membrane by polymorphonuclear leukocytes occurred only until day 3.

Interestingly, the observation of an intense inflammatory reaction spatially coincides with the detection of giant macrophages in the SF. At day 7 histologic evaluation revealed only focal neutrophilic infiltrates of the synovial membrane that are consistent with mild and focal synovitis, which correlates with the non-inflammatory SF white-cell count observed at this time point. These findings suggest that deposition of MSU crystal in the joint space and synovial membrane elicits a strong inflammatory reaction that promotes recruitment of a large number of polymorphonuclear leukocytes to the SF within 24 h. As the inflammatory response temporarily progresses, the synovial membrane becomes hyperplastic and thickened by widespread inflammatory cell infiltrates, resident macrophages in the SF become active and start to phagocyte MSU crystals, and polymorphonuclear leukocytes segregate within the synovial membrane. This reaction is self-limited and by day 7 the majority of MSU crystal deposits have been phagocytized, the SF becomes non-inflammatory, and few leukocytes can be found within the synovial membrane, with only mild synovitis. Visualization of MSU crystals over the cartilage surface was accomplished by gross examination of the articular cartilage but was not shown histologically. This discrepancy can be explained by mechanical loss of the crystals by surgical removal of the extremity, vibration of the band saw and mechanical washing process resulting in MSU-crystal loss [35].

Animal models of human illness provide invaluable tools to understand the basic biological mechanisms for identifying and validating novel molecular targets and pathways involved in the pathogenesis of the disease, and to identify potential therapeutic and preventive agents [45]. The fact that the rabbit knees injected with MSU crystals were the only ones to produce a severe inflammatory synovial response and reproduced the typical US findings of human gout, supports both the internal and external validity of our animal model. The concurrent validity of the animal model was established by demonstrating US-detected abnormalities and the presence of MSU crystals by both polarized light microscopy and histological analysis as the gold standard.

Our study has several limitations. First, in contrast to the systemic and local inflammatory process triggered by the *in-situ* re-crystallization of endogenous MSU crystals in the synovial membrane, our model responded with a local inflammatory reaction after inoculation of exogenous synthetic MSU crystals. Second, clinical parameters such as pain, temperature, swelling, behavioral testing, and disability were not evaluated. Third, we only evaluated intra-articular changes due to MSU-crystal injections within a limited time period (7 days); it would be appropriate to monitor the intra-articular changes for a longer period of time and/or recurrent MSU-crystal injections.

## Conclusions

In conclusion, our rabbit knee model of MSU crystal induced effectively an acute joint inflammatory process, and accurately depicted the early morphostructural changes observed by US during an acute gouty attack. US, SF, and histological analyses provide a working hypothesis: the early presence of deposits and aggregates of MSU crystals is critical in the acute disease phase, challenging the traditional paradigm that this process was limited to the chronic disease stages. The rabbit knee model of MSU crystal-induced arthritis serves as an ideal temporal, spatial, and multimodal platform for further study of the inflammatory process and for detecting structural changes at the joint level during an acute episode of gouty arthritis. Additionally, it affords the opportunity for testing of different pharmacological strategies in the management of gout.

## Additional file

Additional file 1:
**Morphological and birefringence properties of monosodium urate (MSU) crystals were assessed by polarized light (400 ×) and scanning electron microscopy (50,000 ×).**

## Abbreviations

BSA: bright stippled aggregates
DCS: double contour sign
H&E: hematoxylin-eosin
IL: interleukin
MSU: monosodium urate
NALP3: NACHT, LRR and PYD domain-containing protein 3
OMERACT: Outcome Measures in Rheumatology
PBS: phosphate-buffered saline
PD: power Doppler
SF: synovial fluid
US: ultrasound
