# Supplementary material for: Animal model of acute gout reproduces the inflammatory and ultrasonographic joint changes of human gout
Source: Arthritis Res Ther. 2015 Feb 26;17(1):37. doi: 10.1186/s13075-015-0550-4 (PMC4363186; doi:10.1186/s13075-015-0550-4)
Supplement: Supplementary file 1 [file 13075_2015_550_MOESM1_ESM.doc]

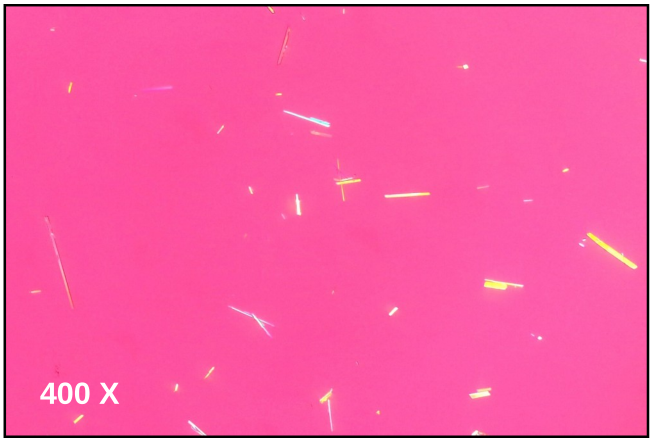
400 X

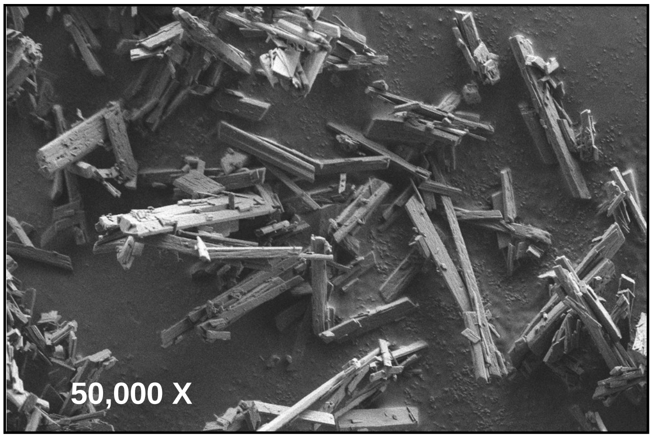
50,000 X
